# Modeling Pulmonary Gas Exchange and Single-Exhalation Profiles of Carbon Monoxide

**DOI:** 10.3389/fphys.2018.00927

**Published:** 2018-07-30

**Authors:** Ramin Ghorbani, Anders Blomberg, Florian M. Schmidt

**Affiliations:** ^1^Department of Applied Physics and Electronics, Umeå University, Umeå, Sweden; ^2^Division of Medicine, Department of Public Health and Clinical Medicine, Umeå University, Umeå, Sweden

**Keywords:** carbon monoxide (CO), pulmonary gas exchange, computational modeling, real-time breath gas analysis, single-exhalation profile, laser absorption spectroscopy

## Abstract

Exhaled breath carbon monoxide (eCO) is a candidate biomarker for non-invasive assessment of oxidative stress and respiratory diseases. Standard end-tidal CO analysis, however, cannot distinguish, whether eCO reflects endogenous CO production, lung diffusion properties or exogenous sources, and is unable to resolve a potential airway contribution. Coupling real-time breath gas analysis to pulmonary gas exchange modeling holds promise to improve the diagnostic value of eCO. A trumpet model with axial diffusion (TMAD) is used to simulate the dynamics of CO gas exchange in the respiratory system and corresponding eCO concentrations for the first time. The mass balance equation is numerically solved employing a computationally inexpensive routine implementing the method of lines, which provides the distribution of CO in the respiratory tract during inhalation, breath-holding, and exhalation with 1 mm spatial and 0.01 s temporal resolution. Initial estimates of the main TMAD parameters, the maximum CO fluxes and diffusing capacities in alveoli and airways, are obtained using healthy population tissue, blood and anatomical data. To verify the model, mouth-exhaled expirograms from two healthy subjects, measured with a novel, home-built laser-based CO sensor, are compared to single-exhalation profiles simulated using actual breath sampling data, such as exhalation flow rate (EFR) and volume. A very good agreement is obtained in exhalation phases I and III for EFRs between 55 and 220 ml/s and after 10 and 20 s of breath-holding, yielding a unique set of TMAD parameters. The results confirm the recently observed EFR dependence of CO expirograms and suggest that measured end-tidal eCO is always lower than alveolar and capillary CO. Breath-holding allows the observation of close-to-alveolar CO concentrations and increases the sensitivity to the airway TMAD parameters in exhalation phase I. A parametric simulation study shows that a small increase in airway flux can be distinguished from an increase in alveolar flux, and that slight changes in alveolar flux and diffusing capacity have a significantly different effect on phase III of the eCO profiles.

## Introduction

Endogenous carbon monoxide (CO) is mainly produced by systemic heme oxygenase and eliminated via respiration. In the healthy, non-smoking population, end-tidal concentrations are in the range 1–3 parts per million (ppm) (Ryter and Choi, 2013). Since CO easily combines with hemoglobin, release and uptake are largely determined by the diffusion properties of the capillary tissue membrane. Given the low water-solubility of CO, the gas exchange occurs almost exclusively in the alveoli, and alveolar CO correlates with blood carboxyhemoglobin (COHb) (Sandberg et al., 2011). However, there is evidence that CO can also arise due to locally induced heme oxygenase, e.g., in airway tissue (Horváth et al., 1998). Moreover, CO has been identified as a cellular signaling molecule (Kim et al., 2006), possibly involved in anti-inflammatory and cytoprotective responses (Ryter and Choi, 2013). In the rapidly evolving field of breath gas analysis, exhaled breath CO (eCO) is thus considered a potential biomarker for non-invasive assessment of oxidative stress and respiratory diseases (Owens, 2010; Amann and Smith, 2013).

A standard eCO measurement typically implies determining a single end-tidal or mixed-breath CO concentration value. Often, analytical devices with low sensitivity, precision and time-resolution, such as electrochemical sensors (Vreman et al., 1993), are employed. From such measurements it is unclear, whether eCO levels outside the healthy population range are due to variations in endogenous blood CO, lung diffusion properties or from exposure to exogenous sources (smoking, air pollution). Also, a potential small contribution from the airways cannot be resolved. These shortcomings have led to ambiguous results in medical studies and shed doubt over the diagnostic value of eCO (Gajdócsy and Horváth, 2010). In clinical practice, the use of eCO is currently limited to the assessment of smoking status (Sandberg et al., 2011) and CO poisoning (Roderique et al., 2015).

Two key factors could improve the diagnostic value of eCO detection. First, novel optical techniques employing mid-infrared lasers have enabled accurate real-time eCO quantification even with compact setups based on tunable diode laser absorption spectroscopy (TDLAS) (Wang and Sahay, 2009), such as those recently developed in our group (Ghorbani and Schmidt, 2017a,b). The precise measurement of single-exhalation profiles with high time-resolution can provide spatial information on the respiratory tract and was recently used to assess oxygen consumption (Ciaffoni et al., 2016) and lung inhomogeneity (Mountain et al., 2018). Second, as has been demonstrated for e.g. exhaled nitric oxide (eNO), carbon dioxide (eCO_2_), ethanol and acetone, significant advances in the interpretation of and information gained from real-time breath data can be achieved by coupling experimental results to mathematical models of gas exchange and physiology (George and Hlastala, 2011; King et al., 2011).

There are two major categories of physiological models developed for pulmonary gas exchange characterization, two-compartment models (Tsoukias and George, 1998; Högman et al., 2000; George et al., 2004) and morphological models (Scherer et al., 1975; Shin and George, 2002). In a two-compartment model, the respiratory tract is usually lumped into two separate partitions, the conducting airways and the alveolar region. It is assumed that the gas is perfectly mixed in each compartment. In a more realistic picture, morphological models consider the dichotomous branching structure of the lung and more comprehensive transport mechanisms including axial diffusion to model the mixing of gases in the bronchial tree (Paiva and Engel, 1987; Shin and George, 2002; Van Muylem et al., 2003). In such models, the alveolar compartment is distributed axially over a short distance with rapidly increasing cross-sectional area. The gas exchange is usually characterized by the maximum gas fluxes from the airways (including the conducting airways and the respiratory bronchioles) and the alveoli, and their corresponding diffusing capacities. In both types of models, tissue and blood layers surrounding the compartments can be added in case of a highly water-soluble biomarker, tissue production or perfusion-limited gas exchange (Tsoukias and George, 1998; Karamaoun et al., 2016). Compared to two-compartment models, the morphological approach is normally better suited to simulate single-exhalation profiles. Bronchial and pulmonary blood circulations are of little importance when modeling the diffusion-limited CO exchange. However, given the expected large concentration gradient between the alveolar and airway region, axial diffusion is assumed to play a significant role.

In this work, carbon monoxide gas exchange in the respiratory system is modeled for the first time, by employing a morphological model based on a trumpet-shaped representation of the lung. The physiological parameters used in the simulations are estimated from literature data. Time- and space-resolved CO distributions in the lung, and corresponding single-exhalation profiles, are calculated for various exhalation flow rates and for breath-holding. The simulations are verified by comparison to measured expirograms from two healthy non-smokers obtained using well-controlled online breath sampling and real-time breath gas analysis employing a home-built CO sensor. The dependence of end-tidal concentration and CO elimination rate on the exhalation flow rate is investigated and compared to eNO. Typical axial concentration distributions along the respiratory tract are visualized, and the sensitivity of the model-predicted expirogram shapes to changes in the main TMAD parameters is scrutinized. Implications for eCO analysis, CO physiology, and disease diagnosis are discussed.

## Materials and methods

### Trumpet model and gas exchange equation

**Figure 1:**
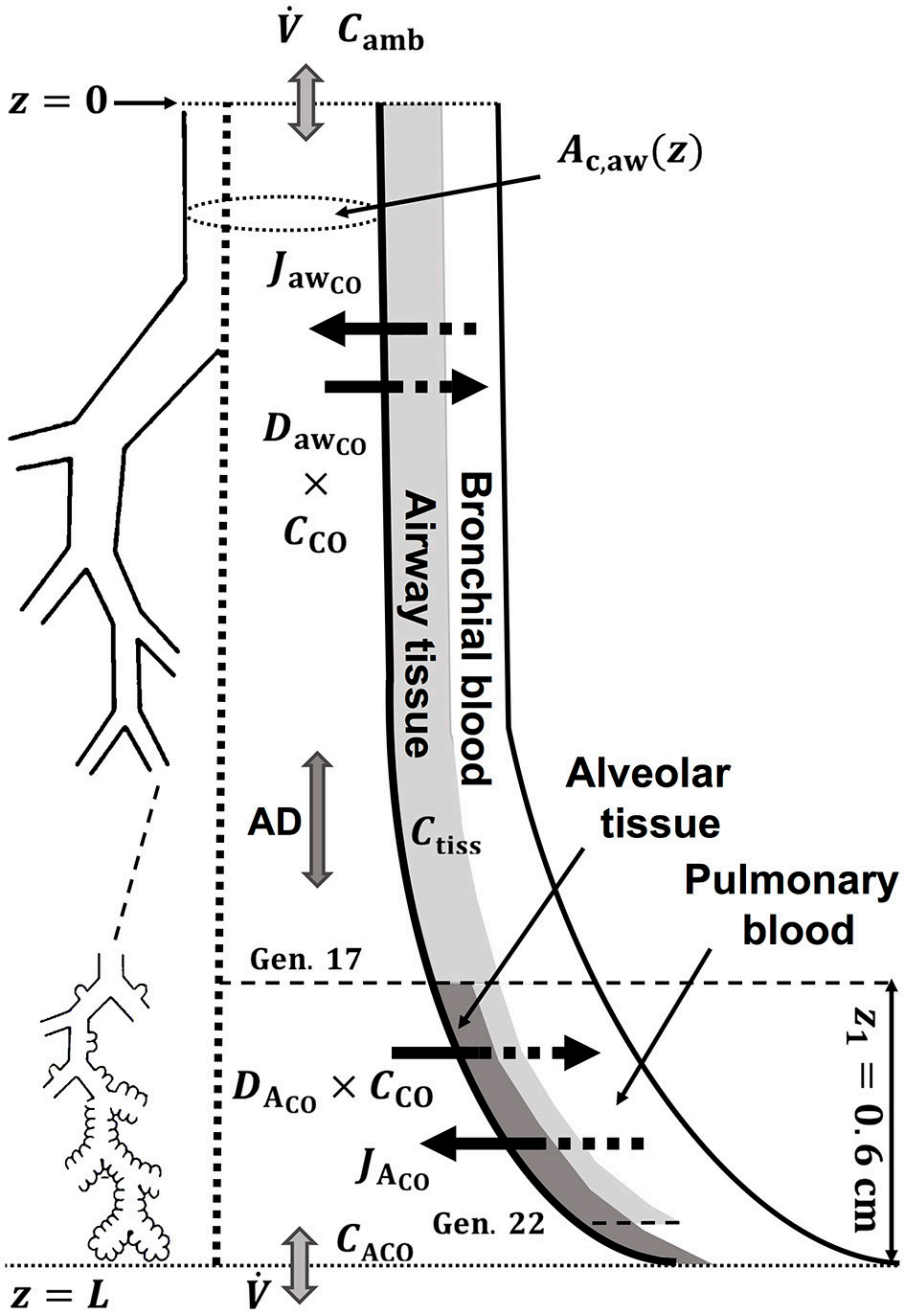
Schematic drawing of the trumpet shape (right) representing the bifurcating structure of the lungs (left) specified by Weibel's anatomical data (Weibel, 1963; Karamaoun et al., 2016). Airway and alveolar gas exchange parameters are indicated. AD, axial diffusion; *C*_tiss_, airway tissue CO concentration; *C*_ACO_, alveolar CO concentration.

A schematic drawing of the one-dimensional trumpet representation of the airway system used in the TMAD adapted from Shin et al. (Shin and George, 2002) is shown in Figure 1. The morphology is based on Weibel's symmetric lung model (Weibel, 1963; Karamaoun et al., 2016), where the human respiratory tract spreads over a bifurcating structure with 24 generations from trachea to alveoli. The system includes the conducting airways (generations 0–16) and the alveolar region (generations 17–23), which consists of respiratory bronchioles (generations 17–22) increasingly interrupted by alveoli. Therefore, the alveolar contribution to CO exchange occurs only between generations 17 and 23, whereas there can be an airway contribution up to the generation 22. The trumpet is considered a rigid, time-independent structure, where the inspired and expired CO molecules are axially transported by the convective bulk flow (reversed after inhalation) and by

(1) $$\begin{array}{l} \left\{A_{\text{c,aw}}\left(z\right)+\left[\frac{N_{\text{alv}}\left(z\right)}{N_{\text{t}}}\right]A_{\text{c,A}}\right\}\frac{\text{d}C_{\text{CO}}}{\text{d}t}=-\overset{\cdot }{V}\frac{\text{d}C_{\text{CO}}}{\text{d}z}+D_{\text{CO,air}}\frac{\text{d}}{\text{d}z}\left[A_{\text{c,aw}}\left(z\right)\frac{\text{d}C_{\text{CO}}}{\text{d}z}\right]+\left({J^{\prime }}_{\text{awCO}}-{D^{\prime }}_{\text{awCO}}C_{\text{CO}}\right)\left[1-\frac{N_{\text{alv}}\left(z\right)}{N_{\text{max}}}\right]\\ \;+\left({J^{\prime }}_{\text{ACO}}-{D^{\prime }}_{\text{ACO}}C_{\text{CO}}\right)\left[\frac{N_{\text{alv}}\left(z\right)}{N_{\text{t}}}\right]. \end{array}$$

gas-phase diffusion. Throughout the entire structure, the model allows for uptake and release of CO across the tissue membrane, representing diffusion into and production by tissue/blood, respectively. The radial net flux at each position in the lung is thus proportional to the prevailing gradient between the partial pressure of CO in the gas phase and in tissue/blood. Due to the low water-solubility of CO, a significant coupling between water vapor concentration, temperature and CO concentration is not expected, and there is no difference in this respect between inspiration and expiration in the model.

Writing the mass balance for a differential control volume in this TMAD model yields an unsteady-state hyperbolic-parabolic partial differential equation for the gaseous CO concentration in the trumpet. The equation includes terms for advection, axial diffusion and the CO production and adsorption rates in the airways and alveoli, and is given by Shin and George (2002)

Here, $\overset{\cdot }{V}$ is the volumetric flow rate ($\overset{\cdot }{V}$_I_ during inhalation and $\overset{\cdot }{V}$_E_ during exhalation), *N*_alv_(*z*) is the number of alveoli per unit axial distance (non-zero only in the alveolar region), *N*_t_ is the total number of alveoli, *N*_max_ is the maximum number of alveoli at any axial position, *A*_c,A_ the total cross-sectional area of the alveolar compartment, *A*_c,aw_ the cross-sectional area of the airway compartment, and *D*_CO,air_ represents the molecular diffusivity of CO in air. The main physiological parameters characterizing the gas exchange are the maximum volumetric fluxes of CO per unit axial distance in airways and alveoli, *J'*_awCO_ and *J'*_ACO_, respectively, and the corresponding diffusing capacities of CO per unit axial distance, *D'*_awCO_ and *D'*_ACO_. These parameters are considered time-independent and uniformly distributed per unit volume. As the number of alveoli per unit axial distance increases with z in the alveolar region, the airway and alveolar contributions on the right-hand-side of Equation (1) progressively decrease and increase, respectively. A single, constant flow rate is assumed along the trumpet shape and possible velocity gradients of the gas perpendicular to the advection are neglected. The ratios between the maximum fluxes and diffusing capacities are considered to represent the airway tissue and alveolar concentrations at equilibrium conditions (Shin and George, 2002).

**Figure 2:**
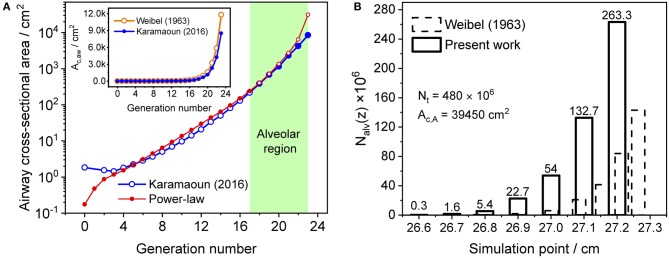
**(A)** Airway cross-sectional area as a function of generation number for the rescaled morphometric data (blue markers), and obtained from the power-law relation with *m* = 2 (red markers). Solid markers show the values considered in the model. Inset: comparison between Weibel's original (orange) and the rescaled (blue) morphometric data. The shaded area indicates the alveolar region. **(B)** Distribution of the number of alveoli per unit axial distance used in Weibel (1963) (dashed bars) and in the current study (solid bars). The alveolar cross-sectional area follows the same distribution function.

The cross-sectional area of the airway compartment can be represented by the power-law relation (Shin et al., 2005)

(2) $$\begin{array}{l} A_{\text{c,aw}}\left(z\right)=A_{\text{c,1}}{\left(\frac{L-z}{z_{1}}\right)}^{-m}, \end{array}$$

where *z*_1_ is the length of alveolar region (0.6 cm beyond generation 17), *A*_c,1_ is the cross-sectional area of the airways at generation 17 and *L* is the total length of the trumpet structure. Equation (2) best matches Weibel's anatomical data for *m* equal to two (Shin et al., 2005). In this work, the airway cross-sectional area is rescaled for a total airspace volume of 3,700 ml (Karamaoun et al., 2016). Figure 2A shows the airway cross-sectional area as a function of generation number for the rescaled morphometric data points (blue), and those obtained from the power-law relation with *m* = 2 (red). The inset depicts a comparison between Weibel's original data (orange hollow markers) and the rescaled values (blue markers). Since the power-law relation overestimates the airway cross-sectional area in the alveolar region, it is here used only up to generation 17 (red solid markers), and the rescaled data are considered directly at generations 18–23 (blue solid markers).

The total number of alveoli in the human lung is considered to be 480 × 10^6^ (Ochs et al., 2004) and axially distributed over the alveolar region based on the rescaled distribution function of the alveolar cross-sectional area in Karamaoun et al. (2016). Figure 2B shows a comparison between the spatial distributions of the alveoli in Weibel (1963) and in the current study. In contrast to Weibel's original data, the relative distribution of alveoli is shifted toward higher generations, which causes the alveolar term to contribute more to the total CO. However, although the total number of alveoli has increased by a factor of 1.6, the functions *N*_alv_(*z*)/*N*_max_ and *N*_alv_(*z*)/*N*_t_ in Equation (1) change only slightly when using the new data. Thus, in terms of modeling, the difference in absolute number and axial distribution of the alveoli depicted in Figure 2B plays a minor role (1% difference in *J*_ACO_ and *D*_ACO_). The rescaling of the alveolar cross-sectional area has a significantly larger effect on the simulated CO concentration. The physical and anatomical parameters used in the present model implementation are listed in Table 1.

**Table 1:** Parameters defining the trumpet model with axial diffusion used in this work.

**Parameter**	**Value**	**Unit**	**References**
*D*_CO,air_	0.21	cm^2^/s	Cussler, 1997
*D*_NO,air_	0.23	cm^2^/s	Shin and George, 2002
*L*	27.2	cm	Karamaoun et al., 2016
*A*_c,1_	217	cm^2^	Karamaoun et al., 2016
*A*_c,A_	39444	cm^2^	Karamaoun et al., 2016
*N*_max_	263.3E+6	–	Ochs et al., 2004; Karamaoun et al., 2016
*N*_t_	480E+6	–	Ochs et al., 2004
*z*_1_	0.6	cm	Karamaoun et al., 2016

### Model solution

The gas exchange equation is solved numerically employing the method of lines. Finite difference approximations of first and up to second order are used to discretize the temporal and the spatial dimensions, respectively. Spatially, the equation is discretized into *n* sections and *n*+1 grid points using a one-sided, upwind approximation scheme with respect to the direction of convective bulk flow during inhalation and exhalation. The temporal dimension utilizes a backward Euler scheme. The discretized version of Equation (1) appears in the form of a tridiagonal system of algebraic equations for grid points. An implicit Euler integration method, which is unconditionally stable, takes care of the stiffness of the equation. The first grid point represents the mouth, where the exhaled CO concentrations are extracted. The mouth grid point is set to ambient air CO during inhalation, and its CO gradient is equal to zero during breath holding and exhalation (d*C*/d*z* = 0). At the last grid point, which serves as the end of the alveolar region, the axial CO gradient is zero at all times. The actual inhaled ambient air CO concentration, the average inhalation and exhalation flow rates (IFR and EFR), and the average of the inspired and expired air volumes are recorded in the experiments during breath sampling and serve as model input parameters. The mathematical details of the solution and boundary conditions are outlined in Appendix A.

A grid size of 0.1 cm and a time step of 0.01 s were chosen to achieve an appropriate spatial and temporal resolution of the CO distribution along the respiratory tract. Although the number of coupled algebraic equations increases with decreasing grid size, the numerical procedure implemented in MATLAB is fast and computationally inexpensive. A typical simulation for inspired and expired volumes of 800 ml and inhalation and exhalation flow rates of 150 ml/s takes <1 s. For a given tidal volume, the computational time naturally increases with decreasing inhalation and exhalation flow rates.

### Estimation of physiological parameters

Since alveolar and airway CO gas exchange is modeled for the first time, reference values for the four main physiological parameters cannot be found in the literature. Therefore, initial TMAD parameters for systemic CO elimination during normal tidal breathing are estimated for the healthy population based on available anatomical, tissue, and blood properties (Table 2).

**Table 2:** Parameters used for the initial estimation of the maximum fluxes and diffusing capacities of carbon monoxide in the airways and the alveolar region.

**Parameter**	**Range**	**Chosen value**	**Unit**	**References**
**AIRWAYS**				
*A*_Maw_	–	9,100	cm^2^	Shin and George, 2002
d*x*_aw_	20–100	20	μm	Shin and George, 2002; George and Hlastala, 2011
**ALVEOLAR REGION**				
*A*_MA_	9.7E+5 – 1.94E+6	1.30E+6	cm^2^	Shields et al., 2009
*K*_CO_ (37°C)	–	2.15E-5	cm^2^.min^−1^.atm^−1^	Parent, 1992
d*x*_A_	0.3–4	0.6	μm	Shields et al., 2009
**BLOOD**				
COHb	0.45–0.67	0.56	% saturation	Wang, 2001
mcapO_2_Hb	96–98	97	% saturation	Collins et al., 2015
mcap*P*_O2_	85–95	90	mmHg	Barrett et al., 2010
*M*	200–245	220	–	Shields et al., 2009

In general, using Fick's law of diffusion, the net flux of CO (pl/s) across a planar tissue membrane can be written as the product of a diffusing capacity *D*_CO_ (pl.s^−1^.ppb^−1^) and the partial pressure difference (ppb) of the gas across the membrane,

(3) $$\begin{array}{l} J_{\text{CO}}=D_{\text{CO}}\cdot \left(\text{mcap}P_{\text{CO}}-C_{\text{A}}\right), \end{array}$$

where mcap*P*_CO_ is the mean pulmonary capillary partial pressure of CO and *C*_A_ is the alveolar partial pressure of CO (both in ppb). In this work, parts per billion (ppb) and ppm refer to mole fractions (nmol.mol^−1^ and μmol.mol^−1^, respectively). Using a morphometric approach, the diffusing capacity, which was previously shown to be equivalent for CO uptake and elimination (Coburn, 2013), can be expressed in terms of the membrane area *A*_M_, the permeation coefficient for CO in lung tissues at 37°C, *K*_CO_, and the membrane thickness d*x*, as (Parent, 1992)

(4) $$\begin{array}{l} D_{\text{CO}}=\frac{10^{4}}{60}\frac{A_{\text{M}}\cdot K_{\text{CO}}}{\text{d}x}. \end{array}$$

The mean pulmonary capillary partial pressure of CO can be calculated using the Haldane equation (Coburn, 2013),

(5) $$\begin{array}{l} \text{mcap}P_{\text{CO}}=\frac{10^{9}}{760}\frac{\left[\text{COHb}\right]\cdot \left[\text{mcap}P_{{\text{O}}_{\text{2}}}\right]}{\left[\text{mcap}{\text{O}}_{\text{2}}\text{Hb}\right]\cdot M}, \end{array}$$

where mcap*P*_O2_ is the mean pulmonary capillary partial pressure of O_2_, mcapO_2_Hb is the mean pulmonary capillary oxyhemoglobin, COHb is the pulmonary capillary carboxyhemoglobin level, and *M* is the Haldane equilibrium constant for the COHb formation reaction.

Using the healthy non-smoker parameter values for the alveolar region in Table 2, the alveolar diffusing capacity can be estimated to around 7,700 pl.s^−1^.ppb^−1^, and mcap*P*_CO_ is 3,100 ppb. This is consistent with the experimentally observed end-tidal CO levels in the healthy population (1–3 ppm). The total maximum volumetric flux of CO across the alveolar tissue membrane, *J*_ACO_, which occurs when *C*_A_ = 0, i.e.,

(6) $$\begin{array}{l} J_{\text{ACO}}=D_{\text{ACO}}\cdot \text{mcap}P_{\text{CO}}, \end{array}$$

then yields a value of 2.4 × 10^7^ pl/s. The constant factors on the right hand sides of Equations (4, 5) are due to unit conversion.

With the airway wall thickness and surface area given in Table 2, Equation (4) predicts an airway diffusing capacity, *D*_awCO_, of around 1.6 pl.s^−1^.ppb^−1^. Mean pulmonary and bronchial capillary partial pressures of CO can be assumed equal, but the blood volume flowing in proximity of the airways is a factor of 10 smaller than the pulmonary blood volume (~1 vs. ~10% of total systemic circulation; Staub and Dawson, 1996; Woo and Szmuszkovicz, 2009). Thus, using Equation (6), the total maximum volumetric flux of CO in the airways, *J*_awCO_, is about 500 pl/s. The estimated TMAD parameter values are compiled in Table 3.

**Table 3:** Estimated main physiological TMAD parameters for healthy non-smokers.

**Parameter**	**Estimated value**	**Units**
**AIRWAYS**		
*J*_awCO_	500	pl/s
*D*_awCO_	1.6	pl.s^−1^.ppb^−1^
**ALVEOLAR REGION**		
*J*_ACO_	2.4E+7	pl/s
*D*_ACO_	7,700	pl.s^−1^.ppb^−1^

### Simulation of single-exhalation profiles

**Figure 3:**
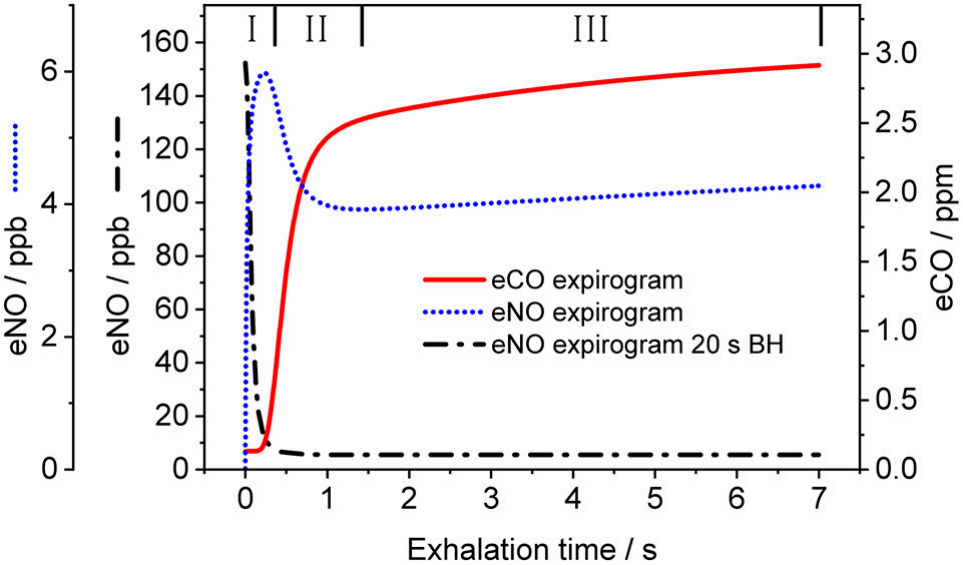
Simulated CO (solid line) and NO (dotted line) exhalation profiles based on the estimated parameters given in Table 3 and the NO parameters in Shin and George (2002), respectively. An eNO profile after 20 s breath-holding (dashed-dotted line) is also shown. IFR and EFR were 200 ml/s and inhaled/exhaled volume was 1,400 ml. The three exhalation phases are indicated by Roman numerals. Assumed inhaled CO and NO concentrations were 130 ppb and zero, respectively.

Figure 3 shows a representative CO exhalation profile (solid line) predicted by the TMAD model for the estimated parameters, an inhalation/exhalation flow rate of 200 ml/s, and an inhaled/exhaled volume of 1,400 ml. The three phases of exhalation (indicated by Roman numerals) can clearly be distinguished. In the model, phase I represents the conducting airways, which, for healthy subjects, contribute little to eCO in addition to ambient air CO. Phase II is the transition between airways and alveoli, and phase III constitutes the CO from the alveolar region.

For comparison, Figure 3 also shows NO exhalation profiles simulated with the TMAD for the same flow rate and volume without BH (dotted line) and after 20 s of BH (dashed-dotted line). The model parameters for NO were taken from Shin and George (2002), i.e., 640 pl/s for *J*_awNO_, 4.2 pl.s^−1^.ppb^−1^ for *D*_awNO_, 3,638 pl/s for *J*_ANO_, and 1,467 pl.s^−1^.ppb^−1^ for *D*_ANO_. Ambient air CO and NO concentrations were 130 ppb and zero, respectively. There is a good agreement between the NO expirograms obtained in this work and those presented in Shin and George (2002). In contrast to eCO, which mostly stems from alveolar gas exchange, eNO shows a peak in phase I due to NO production in the conducting airways. This phase I contribution becomes larger after breath-holding, as tissue NO had time to enrich the gas residing in the airways.

### Laser-based CO sensor and breath sampler

The experimental real-time eCO data were recorded using a compact TDLAS sensor and an online breath sampling system described in detail by Ghorbani et al. (Ghorbani and Schmidt, 2017b). The device makes use of an external-cavity quantum cascade laser, a low-volume multipass sample cell and wavelength modulation spectroscopy to enable CO detection down to 9 ppb at 0.14 s acquisition time and a precision of 2 ppb. Sample pressure and temperature during CO analysis in the multipass cell were 100 Torr and close to room temperature (ca. 23°C), respectively. Concentrations in exhaled breath and ambient air were quantified with high selectivity and free from interference due to water vapor or other volatile compounds. Breath carbon dioxide could also be measured. The breath sampler consisted of a buffer tube made of Teflon and an inline flow meter and capnograph. A Teflon mouthpiece and an antibacterial filter were mounted at the sampler inlet. At the exit of the buffer tube, a two-way valve regulated the inhalation and exhalation routes. An orifice at the exit port was used to facilitate confining the EFR to a narrow range. A portion of the exhaled breath was sampled from the buffer tube and led to the TDLAS sensor with a flow rate of 50 ml/s. Audiovisual indicators implemented in a LabVIEW computer interface helped subjects to maintain a certain EFR and breathing frequency. By direct comparison between eCO_2_ measured by TDLAS and capnography, it was established that the exhalation profiles are recorded in real-time without instrumental signal distortion (Ghorbani and Schmidt, 2017b).

### Study protocol

Two healthy non-smokers provided mouth-exhaled breath samples at three different exhalation flow rates in the range 55–220 ml/s and after 10 or 20 s of breath-holding, respectively. Subject 1 (male, 41 years) and subject 2 (female, 27 years) had body mass indices of 25 and 20 kg/m^2^, respectively. For each breathing maneuver, the subjects took a relaxed sitting position and provided a sequence of 4–5 breath cycles, of which one was later chosen as representative. The inhalation/exhalation flow rate and volume (around 800 and 1,050 ml for subjects 1 and 2, respectively) were continuously recorded with the breath sampler. Ambient air was sampled and analyzed during inhalation. The study protocol was approved by the Regional Ethical Review Board at Umeå University (2017/306-31). All subjects gave written informed consent in accordance with the Declaration of Helsinki.

## Results

### Comparison of simulated and experimental CO exhalation profiles

**Figure 4:**
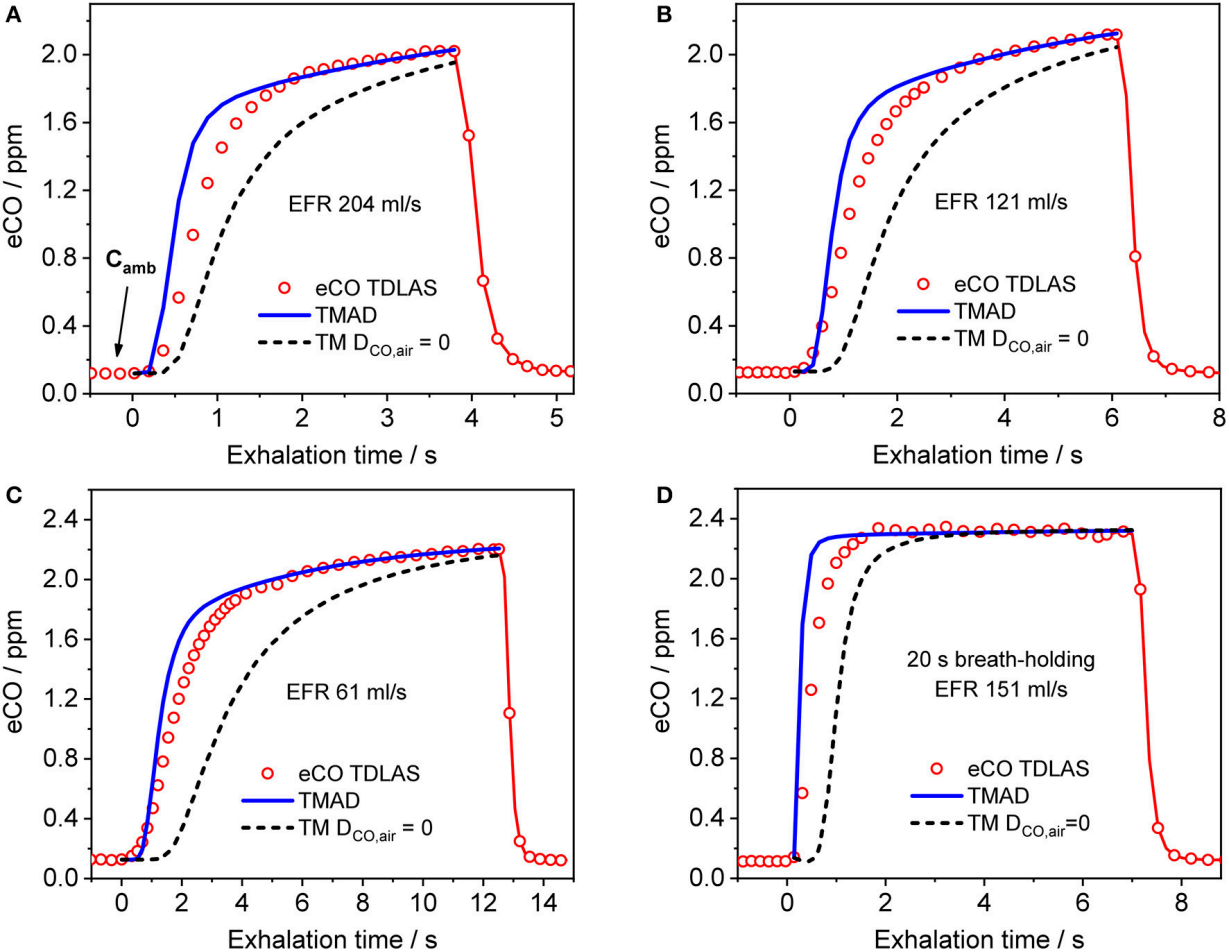
Comparison between measured (hollow markers) and model-predicted (solid lines) eCO profiles for different EFRs **(A–C)**, and 20 s breath-holding **(D)** for subject 1. For clarity, only every 2nd experimental data point is shown in phase III of the eCO profiles in **(B,D)**, and every 3rd data point is shown in phase III of the eCO profile in **(C)**. Corresponding simulations without axial diffusion (short-dashed lines) are shown in **(A–D)**. EFR, exhalation flow rate.

**Figure 5:**
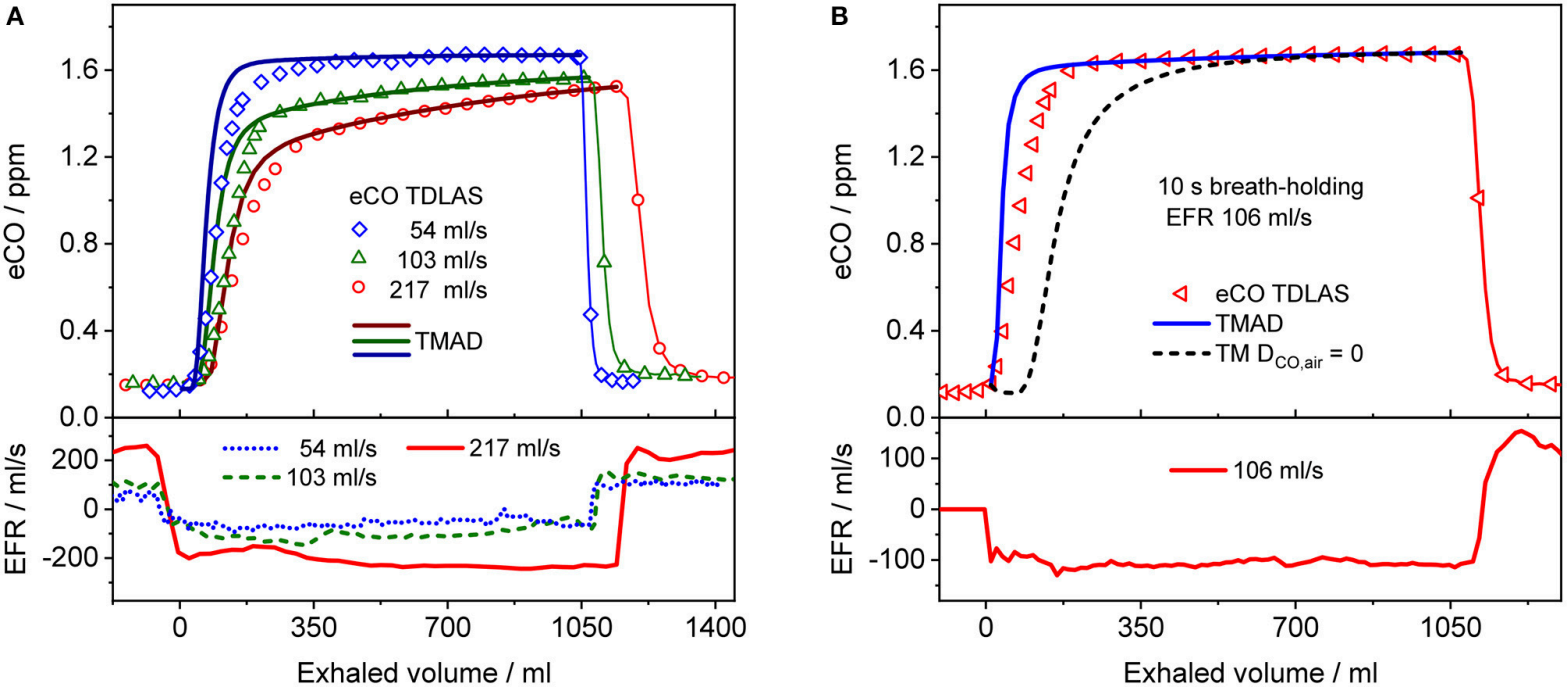
Comparison between measured (hollow markers) and model-predicted (solid lines) eCO profiles for different EFRs **(A)**, and 10 s breath-holding **(B)** for subject 2. For clarity, only every 2nd, 4th, and 7th experimental data point is shown in phase III of the eCO profiles corresponding to EFRs of 217, 103, and 54 ml/s in **(A)**, and every 4th data point is shown in phase III of the eCO profile in **(B)**. A corresponding simulation without axial diffusion (short-dashed line) is shown in **(B)**. Lower panels present corresponding real-time EFR data recorded by the breath sampler.

Figure 4 presents a comparison between expirograms measured with the laser-based CO sensor (markers) and those predicted by the TMAD model (solid lines). The data is from subject 1 for EFRs of 204, 121, and 61 ml/s (Figures 4A–C) and a 20-s breath-holding maneuver (Figure 4D). A similar comparison between measured (markers) and TMAD-predicted (solid lines) expirograms is shown for subject 2 in Figure 5. The data are displayed as a function of exhaled volume for EFRs of 217, 103, and 54 ml/s (Figure 5A) and a 10-s breath-holding maneuver (Figure 5B). Moreover, the real-time EFR data measured by the breath sampler during exhalation is presented in separate panels. Both figures display additional simulations (short-dashed lines) performed with the same TMAD parameters, but without the effect of axial diffusion.

The average of the measured real-time data for the flow rates $\overset{\cdot }{V}$_I_ (IFR) and $\overset{\cdot }{V}$_E_ (EFR), the average of the inhaled and exhaled air volumes (*V*) and the ambient air CO concentration were used as model input parameters. Ambient CO was determined during inhalation, as indicated in Figure 4A. Time zero was determined from the start of the exhalation as recorded by the flow meter, and considering a slight instrumental time delay between flow and eCO measurements. For each subject, first the airway TMAD parameters were determined by adapting the simulation to the first few experimental data points of the BH curve (Figures 4D, 5B), which represent the portion of the exhaled breath (first ~0.2 s) that resided in the conducting airways. The alveolar TMAD parameters were then slightly refined with respect to the initial estimates given in Table 3, to match the individual expirograms. The experimental breath sampling data, including end-tidal CO_2_, and the final TMAD parameters obtained for subjects 1 and 2 are summarized in Tables 4, 5, respectively.

**Table 4:** Physiological model parameters and experimental respiratory data used in the TMAD simulations presented in Figure 4.

							Respiratory data^*^				
	***J*_awCO_**	***D*_awCO_**	***J*_ACO_**	***D*_ACO_**	$C_{\text{CO,ET}}^{*}$	***C*_ACO_**	***$\overset{\cdot }{V}$*_I_**	***$\overset{\cdot }{V}$*_E_**	***V***	***etCO_2_***	***C*_amb_**
	**pl/s**	**pl.s**^−1^**.ppb**^−1^	**pl/s**	**pl.s**^−1^**.ppb**^−1^	**ppb**	**ppb**	**ml/s**	**ml/s**	**ml**	**%**	**ppb**
*A*	220	1.6	2.05E+7	8,800	2,020	2,330	152	204	770	6.0	119
*B*	220	1.6	1.76E+7	7,400	2,118	2,378	121	121	726	5.9	130
*C*	220	1.6	1.65E+7	7,100	2,201	2,324	108	61	765	6.2	125
*D*	220	1.6	1.44E+7	6,100	2,297	2,360	209	151	1,041	6.5	112

The airway parameters and tissue concentration (C_tiss_ = 220/1.6 ≈ 140 ppb) were determined from the first points of the BH profile (Figure 4D). C_CO,ET_, end-tidal CO concentration; V, average of inhaled and exhaled volumes; etCO_2_, end-tidal CO_2_ concentration.

* *Directly measured parameters. All other parameters are derived from the model*.

**Table 5:** Physiological model parameters and experimental respiratory data used in the TMAD simulations presented in Figure 5.

							Respiratory data^*^				
	***J*_awCO_**	***D*_awCO_**	***J*_ACO_**	***D*_ACO_**	$C_{\text{CO,ET}}^{*}$	***C*_ACO_**	***$\overset{\cdot }{V}$*_I_**	***$\overset{\cdot }{V}$*_E_**	***V***	***etCO_2_***	***C*_amb_**
	**pl/s**	**pl.s**^−1^**.ppb**^−1^	**pl/s**	**pl.s**^−1^**.ppb**^−1^	**ppb**	**ppb**	**ml/s**	**ml/s**	**ml**	**%**	**ppb**
*A*	240	1.6	1.65E+7	10,000	1,518	1,650	241	217	1,087	4.9	154
*A*	240	1.6	1.18E+7	7,250	1,560	1,628	122	103	1,064	5.4	162
*A*	240	1.6	1.15E+7	6,700	1,658	1,716	52	54	1,041	5.9	132
*B*	240	1.6	1.05E+7	6,100	1,671	1,721	114	106	1,061	5.8	112

The airway parameters and tissue concentration (C_tiss_ = 240/1.6 ≈ 150 ppb) were determined from the first points of the BH profile (Figure 5B). C_CO,ET_, end-tidal CO concentration; V, average of inhaled and exhaled volumes; etCO_2_, end-tidal CO_2_ concentration.

* *Directly measured parameters. All other parameters are derived from the model*.

### Dependence on exhalation flow rate

**Figure 6:**
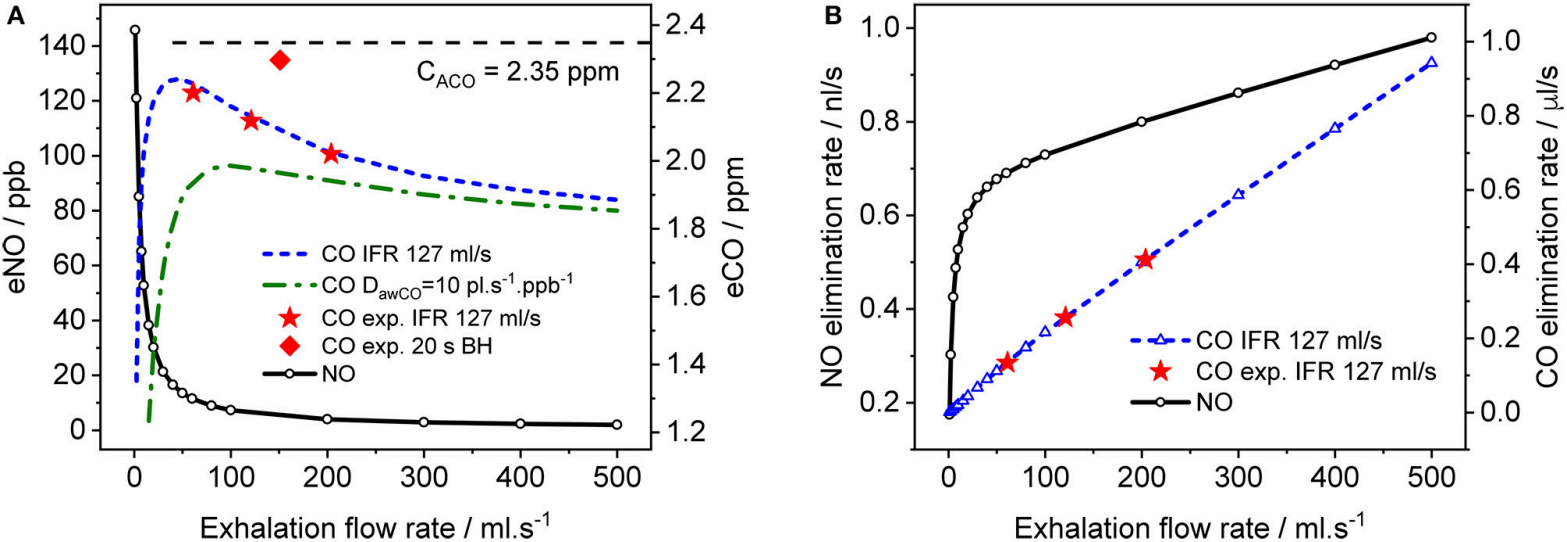
Simulated end-tidal concentrations **(A)** and elimination rates **(B)** as a function of EFR for CO (dashed lines) and NO (solid lines). The average TMAD parameters from Figures 4A–C (i.e., 220 pl/s, 1.6 pl.s^−1^.ppb^−1^, 1.82E+7 pl/s, and 7767 pl.s^−1^.ppb^−1^ for *J*_awCO_, *D*_awCO_, *J*_ACO_, and *D*_ACO_, respectively) are used for CO, and those from Shin and George (2002) for NO. IFR was set to 127 ml/s. Model-predicted alveolar CO concentrations (*C*_ACO_) are indicated by a dashed line in **(A)**. Experimental end-tidal values (Figure 4) for the three EFRs (star markers) and breath-holding (diamond marker) are shown for comparison.

In Figure 6, simulated end-tidal concentrations (Figure 6A) and elimination rates (Figure 6B) of CO (short dashed line) and NO (solid lines) are plotted as a function of EFR. The elimination rate is defined as the product of end-tidal concentration and corresponding EFR. For NO, the parameters in Shin and George (2002) were again used, whereas for CO, the average values of the refined model parameters in Table 4 (A–C) were employed, i.e., 220 pl/s, 1.6 pl.s^−1^.ppb^−1^, 1.82E+7 pl/s, and 7767 pl.s^−1^.ppb^−1^ for *J*_awCO_, *D*_awCO_, *J*_ACO_, and *D*_ACO_, respectively. In Figure 6A, the predicted alveolar CO concentration of 2.35 ppm (the same for all EFRs) is indicated by a dashed line. The experimentally obtained end-tidal CO concentrations from Figure 4 and the corresponding elimination rates are indicated by star markers (Figures 4A–C) and a diamond marker for BH (Figure 4D).

As expected from literature, end-tidal NO is low at high EFRs, but increases dramatically below an EFR of 50 ml/s due to the airway contribution. In contrast, end-tidal CO, first increases with decreasing flow rate down to around 50 ml/s, followed by a sharp decline. All experimentally determined end-tidal CO concentrations are below the model-predicted alveolar levels, even for BH. In order to demonstrate the influence of the airway diffusing capacity, Figure 6A also includes a curve (dashed-dotted line) for a six-fold larger *D*_awCO_ of 10 pl.s^−1^.ppb^−1^. In this case, diffusion of alveolar CO into airway tissue during exhalation increases, leading to a lower curve as a whole and a shift in the peak toward higher EFRs. The simulated NO elimination rate exhibits two separate linear regimes, which is in agreement with literature. The CO elimination rate, on the other hand, is almost linear over the entire simulated EFR range due to the small airway contribution in healthy subjects compared to alveolar CO.

### Spatial-temporal distribution of CO in the respiratory tract

**Figure 7:**
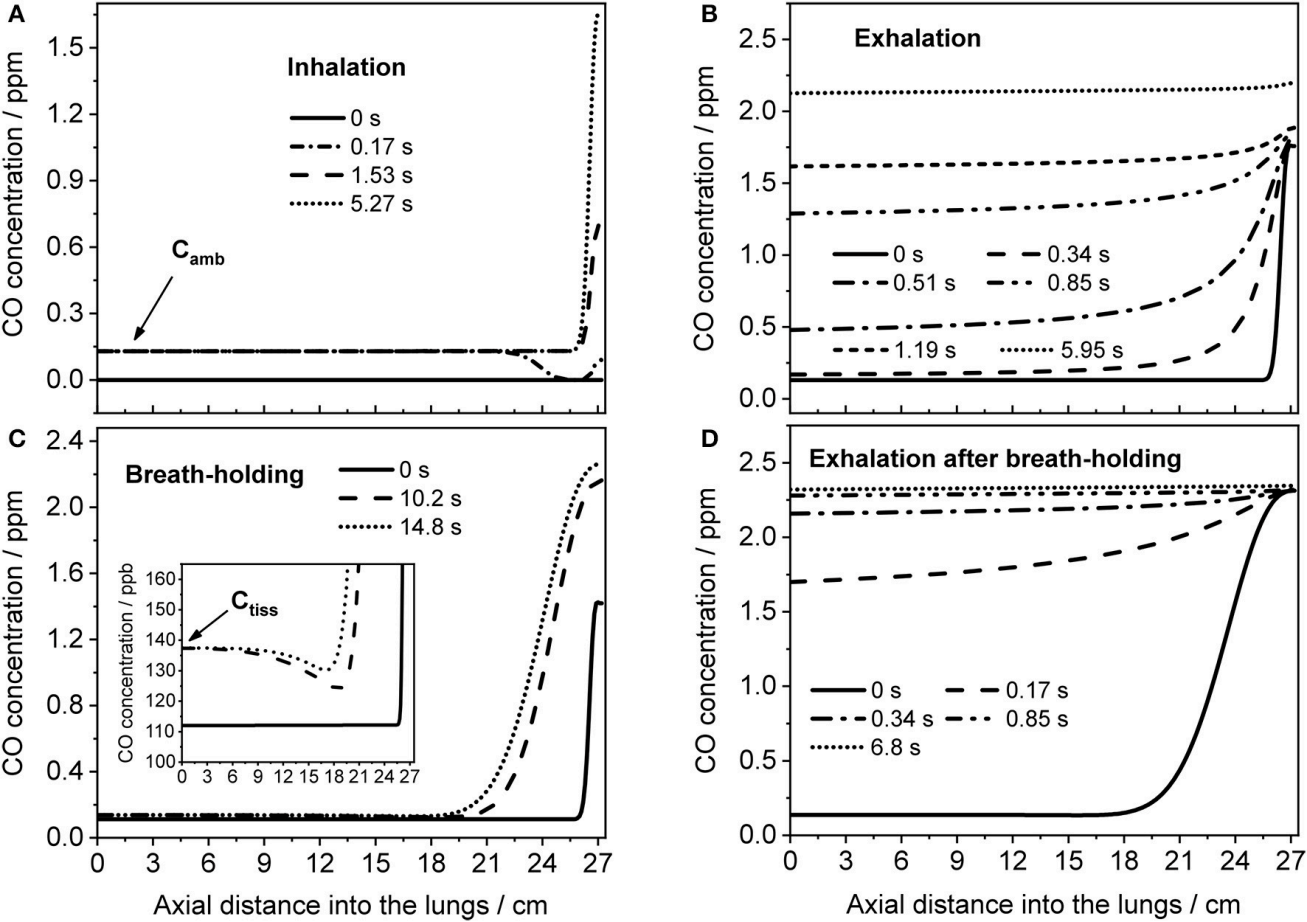
Axial distributions of CO along the respiratory tract for several instances during inhalation **(A)** and subsequent exhalation **(B)** at 121 ml/s (parameters from Figure 4B), and during a 20-s BH maneuver **(C)** followed by exhalation **(D)** at 151 ml/s (parameters from Figure 4D). Inhaled CO and airway tissue concentrations are indicated in **(A,C)**, respectively.

The TMAD model can provide an insight into how the CO concentration distribution in the respiratory tract changes as a function of time and space. This is illustrated in Figure 7, which shows the CO concentration along the trumpet at certain times during inhalation (Figure 7A), breath-holding (Figure 7C) and exhalation (Figures 7B,D). The breathing maneuvers and model parameters correspond to the simulations shown in Figures 4B,D for an EFR of 121 ml/s and a 20-s BH maneuver, respectively. Inhaled CO and airway tissue concentrations are indicated.

Initially, the CO concentration in the respiratory tract is assumed zero. Then, ambient air is inhaled and systemic CO starts to diffuse into the alveolar region from tissue/blood (Figure 7A). The last profile from inhalation is used as initial eCO profile for the exhalation (Figure 7B), where the convective flow forces the alveolar air through the airways, while the alveolar CO concentration is still increasing. If the breath is held for 20 s after inhalation, the model predicts the distributions shown in Figure 7C. Here, axial diffusion is the main transport mechanism, with the steep gradient between alveolar and airway concentrations resulting in CO diffusion toward the airways. Toward the end of exhalation, alveolar and airway CO approach equilibrium with tissue/blood and cease to increase. During exhalation after BH (Figure 7D), there is a steep eCO increase already in phase I, quickly reaching a plateau with close-to-alveolar CO concentrations.

### Dependence of expirogram shape on main TMAD parameters

**Figure 8:**
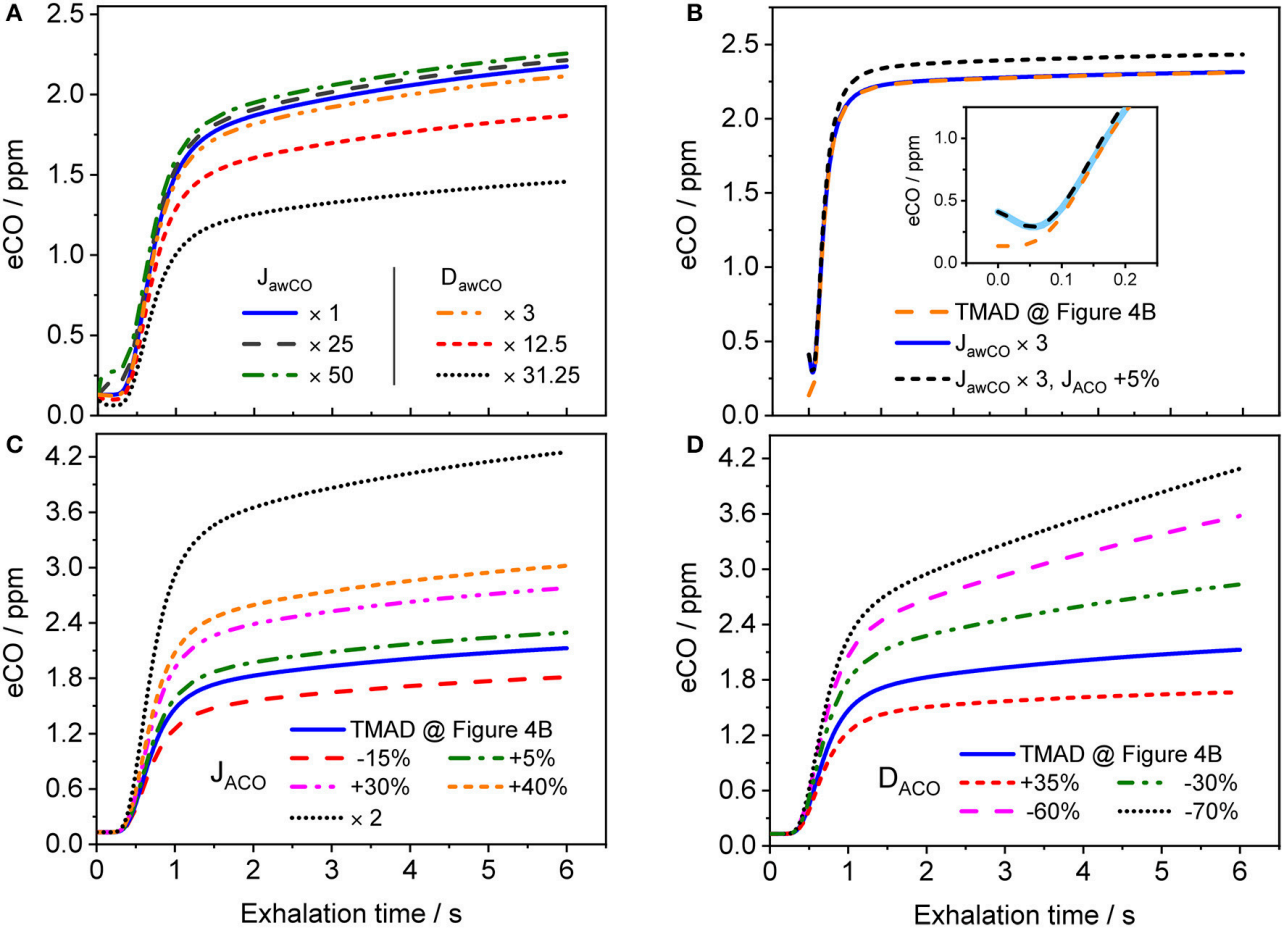
Simulated eCO profiles for variations in **(A)** maximum airway flux or airway diffusing capacity, **(B)** maximum airway and alveolar fluxes simultaneously (including 10 s BH), **(C)** maximum alveolar flux, and **(D)** alveolar diffusing capacity. The inset in **(B)** shows a close-up on the first 0.25 s of the exhalation, with the abscissa in terms of exhalation time. Initial TMAD parameters are similar to those used for the simulation in Figure 4B. The exhalation flow rate and the exhaled volume are 121 ml/s and volume 726 ml, respectively. Parameter units as in the nomenclature.

Figure 8 shows how the shape of a simulated eCO profile (EFR 121 ml/s, volume 726 ml) changes, when the main TMAD parameters are varied separately. Clearly, for tidal breathing, the profile shape is less sensitive to the airway (Figure 8A) than to the alveolar (Figures 8C,D) parameters. A large increase in maximum airway flux is needed to be able to see a phase I (and III) increase in eCO during tidal breathing. A moderate increase in airway diffusing capacity leads to a decrease in all three exhalation phases. There is a noticeable dip in phase I due to diffusion of inhaled CO into airway tissue (Figure 8A). However, a 10 s breath-holding maneuver (Figure 8B) increases the sensitivity to airway parameters, such that a simultaneous three-fold increase in maximum airway flux and a 5% increase in maximum alveolar flux are distinguishable (Figure 8B). Only a small variation in maximum alveolar flux is needed to cause a significant change in the absolute CO level of phase III, but not in its slope (Figure 8C). A change in alveolar diffusing capacity, on the other hand, alters both the absolute concentration and the slope of phase III (Figure 8D).

## Discussion

The present implementation of the TMAD model is robust, fast and provides stable solutions for a wide range of parameter values and breathing maneuvers (Figures 3–6, 8). It is shown for two healthy non-smokers that the simulated CO profiles match the measured expirograms well if the individual experimental input parameters are used and the TMAD parameters are varied slightly around the initial estimates (Figures 4, 5). The sensitivity analysis (Figure 8) confirms that for a given expirogram shape the individual TMAD parameters obtained from comparison are unique within a narrow range (around 1% for the alveolar and 7% for the airway parameters). Axial diffusion plays a crucial role when modeling CO gas exchange, even for exhalation phase III (Figures 4A–D, 5B). Without the effect of axial diffusion, significantly higher alveolar TMAD parameters than the estimates would be required to achieve a good agreement between simulation and experiment.

The maximum alveolar fluxes matching the experimental data (absolute phase III level) are close to the initial estimate (Tables 3–5), as expected given that COHb varies in a narrow range in the healthy, non-smoking population. However, maximum flux and end-tidal CO are higher for subject 1 than for subject 2, while the alveolar diffusing capacities (slope and absolute level of phase III) are similar for each EFR. This yields a higher alveolar CO concentration for subject 1 and indicates a slightly higher COHb level. The absolute values of the alveolar diffusing capacity, which represent an average over the entire exhalation maneuver, are in accordance with the morphological estimate, but considerably higher than the standard *D*_LCO_ data. The main reasons are that the physiological conditions during systemic elimination and tidal breathing are very different from those prevailing in the *D*_LCO_ test, and that morphological models tend to overestimate the diffusing capacity (Hughes and Bates, 2003). As evident from Tables 4, 5, for both subjects, the alveolar parameters (except alveolar CO) as well as end-tidal CO and CO_2_ exhibit an EFR dependence. While *J*_ACO_ and *D*_ACO_ increase with increasing EFR, end-tidal CO and CO_2_ decrease. Variations in eCO_2_ due to hypo- or hyper-ventilation have previously been shown to correlate with eCO (Cavaliere et al., 2009). Here, all measurements have been performed with similar inhalation/exhalation volumes to minimize the effects of breath sampling. The end-tidal eCO_2_ levels vary in a narrow range for each subject and indicate moderate hypoventilation for subject 1 and normoventilation for subject 2 (Cavaliere et al., 2009). The underlying reason for the observed EFR dependence is that the gas residence time in the lung varies with flow rate. In case of CO, a longer residence time brings the CO concentration closer to equilibrium with capillary CO.

Given the perpetual exposure of the airway tissue to ambient and alveolar CO, it is not surprising that the observed tissue concentrations are similar to, or slightly above, ambient CO. For an EFR of 120 ml/s, the sensitivity to the airway parameters is relatively low, since only a small part of the exhaled volume (phase I, part of phase II) exclusively interacts with the airways, and because the airway tissue concentration, although of the same order as tissue NO, is low compared to alveolar CO in healthy non-smokers. The sensitivity increases for low EFRs and BH, due to the longer gas residence time in the respiratory tract.

The discrepancy between experiment and simulation in exhalation phase II (Figures 4, 5) can have several reasons. The model assumes fixed inhalation and exhalation flow rates, while, in practice, although well-controlled by the breath sampler, the flow rate may vary, in particular at the start and end of the maneuver (Figure 5). In addition, the model geometry, e.g., number and distribution of alveoli, cross-sectional areas and the length of the respiratory tract, is only approximate, and may differ between individuals. This includes that the volumes of anatomical dead space and oral/nasal cavities are not reflected in the cross-sectional areas of the compartments. Moreover, not all the gas mixing mechanisms present in the lungs are accounted for in the one-dimensional model. For instance, in reality, the airway cross-sectional area is changing during inhalation and exhalation, and different regions of the lungs are ventilated at different rates.

Exhaled biomarker concentrations may depend on IFR and EFR, if any of the involved gas exchange processes occur on time scales similar to or lower than the residence time of the gas in the respiratory tract. Such processes include gas exchange across the thick airway tissue (as for NO) and interaction with the airway mucus (for highly water soluble gases; Anderson et al., 2003). Figures 4, 5, 6A confirm previous experimental observations (Fritsch et al., 2007; Raiff et al., 2010; Ghorbani and Schmidt, 2017b) that end-tidal CO and CO elimination depend on the exhalation flow rate. At EFRs above 50–100 ml/s (depending on the tissue properties), a decreasing end-tidal CO is observed with increasing EFR, due to the slow gas transfer from capillary blood to alveolar gas limited by the diffusion through the capillary membrane. For lower flow rates, end-tidal CO decreases rapidly with EFR, probably due to diffusion of alveolar CO into airway tissue during exhalation. This indicates that CO gas exchange may not solely be confined to the alveoli. The model-predicted alveolar level is similar for all maneuvers, but close-to-alveolar CO concentrations (still influenced by the finite airway diffusing capacity) can experimentally only be observed after breath-holding. Therefore, end-tidal CO, even after BH, is not necessarily equal to alveolar CO. This has consequences when end-tidal CO is used to estimate COHb or red blood cell lifespan (Furne et al., 2003).

The CO distributions in the respiratory tract visualized in Figure 7 confirm the important role of axial diffusion in driving alveolar CO into the airways, in particular during breath-holding. If the airway tissue concentration is larger than the inhaled ambient CO level, the air in the conducting airways is enriched with CO during BH, until the tissue concentration is reached (inset Figure 7C). The enrichment is less pronounced at axial distances of 12–18 cm into the lung (the end of phase I), where the cross-sectional area starts to increase and diffusion into tissue has the largest effect. Thus, as for NO (Shin et al., 2005), breath-holding could amplify a potential contribution from the conducting airways to be extracted from phase I of an exhalation profile. Figure 8 further illustrates that a slightly elevated airway flux, for example in response to an inflammation or due to external exposure, is more likely resolved using a BH maneuver rather than normal tidal breathing.

Importantly, the fact that the expirogram shape depends uniquely on the individual alveolar TMAD parameters provides the possibility to discriminate, whether an end-tidal CO concentration outside the healthy population range stems from unusual pulmonary diffusion properties or blood CO levels. For example, the eCO profiles shown by dotted lines in Figures 8C,D have similarly elevated end-tidal values (>4 ppm), but their shapes are clearly different. The expirogram in Figure 8C is simulated for a high maximum alveolar flux, as could be caused by a high COHb level due to systemic oxidative stress or smoking, whereas the curve in Figure 8D is simulated for a low alveolar diffusing capacity, as might occur in severe chronic obstructive pulmonary disease (COPD). The strategy to make use of the phase III slope to determine lung diffusion properties was previously proposed for eCO_2_ (Schwardt et al., 1994), but the effect may be more pronounced for CO given the diffusion-limited gas exchange. To distinguish, whether an observed increase in blood CO originates from exogenous or endogenous sources will, however, still be difficult using the current approach.

The benefits of the measurement technique used in this work compared to conventional (end-tidal) eCO analyzers are: (i) very high sensitivity, precision and absolute accuracy, (ii) controlled breath sampling (IFR/EFR, volume, breathing frequency), (iii) simultaneous measurement of eCO_2_ (indication for hypo/hyper-ventilation), (iv) precise real-time exhalation profiles provide more accurate end-tidal CO concentrations and facilitate the interpretation of unusual eCO levels (endogenous production, diffusion, EFR), and (v) by comparison with the gas exchange model, airway CO can be resolved and accurate alveolar CO levels can be predicted that are largely independent of EFR and hypo/hyper-ventilation.

Possible model improvements depend in part on the availability of more accurate morphometric data of the trumpet shape, including dead space volume and cross-sectional areas, and the implementation of more detailed gas mixing mechanisms. The phase I and II agreement could be enhanced by using the actual, instantaneous inhalation and exhalation flow rates measured by the breath sampler, instead of a fixed average value. A more precise determination of the physiological parameters could be achieved by least-squares fitting of simulated to measured expirograms. Future studies should focus on determining the healthy population baseline for the TMAD parameters and on clinical studies with diseased subjects to verify and further investigate the value of the extended eCO analysis.

## Conclusion

A trumpet model incorporating gas-phase axial diffusion was adapted to, for the first time, predict the gas exchange dynamics of carbon monoxide in the airways and alveoli. Simulated single-exhalation profiles were found in good agreement with measured healthy non-smoker expirograms for different exhalation flow rates and breath-holding. Axial diffusion plays a significant role in distributing the CO molecules in the respiratory tract, in particular during breath-holding. Physiological parameters, such as CO fluxes, diffusing capacities and prevailing gas concentrations in the alveolar region and conducting airways, can be determined uniquely from systemic CO elimination and using a single exhalation at constant flow rate. End-tidal CO was found to be lower than alveolar CO, even after breath-holding, and dependent on exhalation flow rate. Results from simulations suggest that the expirogram shape, in particular exhalation phase III, provides means to distinguish between changes in pulmonary diffusion and blood CO. Airway CO can best be resolved after breath-holding. Extended eCO analysis based on real-time measurements and mathematical modeling has the potential to enhance the diagnostic value of eCO and improve the understanding of CO physiology.

## Author contributions

FS and RG made substantial contributions to the conception and design of the paper. RG implemented the mathematical model, carried out the model simulations, performed the experiments and evaluated the raw data. FS and RG analyzed and interpreted the results. RG drafted the manuscript. FS, AB, and RG edited and revised the article. All authors approved the final submission of the document.

### Conflict of interest statement

The authors declare that the research was conducted in the absence of any commercial or financial relationships that could be construed as a potential conflict of interest.

## Appendix A

### Spatial and temporal discretization of the equation

The equation has been discretized spatially and temporally using a flow-direction dependent upwind approximation and an implicit Euler method, respectively. This discretization scheme causes the CO concentration at each grid point and time-step to be dependent on the CO concentrations of that and the neighboring nodes at the next time-step, such that

(A1) $$\begin{array}{l} {C_{j}}^{k}=M_{j}{C_{j-1}}^{k+1}+N_{j}{C_{j}}^{k+1}+P_{j}{C_{j+1}}^{k+1}+J_{j}, \end{array}$$

where *C*_j_^k^ is the CO concentration at node *j* and time-step *k*, and *M*_j_, *N*_j_, *P*_j_, and *J*_j_ are matrices defined separately for inspiration as

(A2) $$\begin{array}{l} M_{j}^{\;insp}=\frac{f_{1}\left(j\right)}{\Delta z^{2}}\left(-\overset{\cdot }{V}\Delta z+f_{2}\left(j\right)\Delta z-f_{3}\left(j\right)\right)\\ N_{j}^{\;insp}=1+\frac{f_{1}\left(j\right)}{\Delta z^{2}}(\overset{\cdot }{V}\Delta z-f_{2}\left(j\right)\Delta z+2f_{3}\left(j\right)\\ \;+D\prime_{awCO}f_{4}\left(j\right)\Delta z^{2}+D\prime_{ACO}f_{5}\left(j\right)\Delta z^{2})\\ P_{j}^{\;insp}=-\frac{f_{1}\left(j\right)}{\Delta z^{2}}f_{3}\left(j\right)\\ J_{j}^{\;insp}=-f_{1}\left(j\right)\left(J\prime_{awCO}f_{4}\left(j\right)+J\prime_{ACO}f_{5}\left(j\right)\right) \end{array}$$

and expiration as

(A3) $$\begin{array}{l} M_{j}^{\;exp}=-\frac{f_{1}\left(j\right)}{\Delta z^{2}}f_{3}\left(j\right)\\ N_{j}^{\;exp}=1+\frac{f_{1}\left(j\right)}{\Delta z^{2}}\left(-\overset{\cdot }{V}\Delta z+f_{2}\left(j\right)\Delta z+2f_{3}\left(j\right)\right)\\ \;+D\prime_{awCO}f_{4}\left(j\right)\Delta z^{2}+D\prime_{ACO}f_{5}\left(j\right)\Delta z^{2})\\ P_{j}^{\;exp}=\frac{f_{1}\left(j\right)}{\Delta z^{2}}\left(\overset{\cdot }{V}\Delta z-f_{2}\left(j\right)\Delta z-f_{3}\left(j\right)\right)\\ J_{j}^{\;exp}=-f_{1}\left(j\right)\left(J\prime_{awCO}f_{4}\left(j\right)+J\prime_{ACO}f_{5}\left(j\right)\right) \end{array}$$

with

(A4) $$\begin{array}{c} f_{1}\left(j\right)=\frac{\Delta t}{A_{c,aw}\left(j\right)+f_{5}\left(j\right)A_{c,A}}\\ f_{2}\left(j\right)=\frac{2A_{c,1}D_{CO,air}z_{1}{\;}^{2}}{{\left(L-j\Delta z\right)}^{3}}\\ f_{3}\left(j\right)=D_{CO,air}A_{c,1}{\left(\frac{L-j\Delta z}{z_{1}}\right)}^{-2}\\ f_{4}\left(j\right)=1-\frac{N_{alv}\left(j\right)}{N_{max}}\\ f_{5}\left(j\right)=\frac{N_{alv}\left(j\right)}{N_{t}}. \end{array}$$

The equations for expiration, but with $\overset{\cdot }{V}$ = 0, are also used to simulate the breath-holding maneuvers. It is to be noted that $\overset{\cdot }{V}$ takes the positive sign during inhalation and negative sign during exhalation.

### Boundary and initial conditions

The boundary conditions for solving the mass balance equation are similar to those used in Shin and George (2002), except that the ambient air concentration is not set to zero but has a finite value equal to the inhaled ambient air CO determined experimentally.

Boundary conditions for inspiration:

(A5) $$\begin{array}{lll} z & = & 0:C\left(0,t\right)={C_{0}}^{k}=C_{amb} \end{array}$$

(A6) $$\begin{array}{lll} z & = & L:C\left(L,t\right)=\frac{\partial {C_{n+1}}^{k}}{\partial z}=0\to {C_{n+2}}^{k}={C_{n}}^{k} \end{array}$$

Boundary conditions for expiration:

(A7) $$\begin{array}{lll} z & = & 0:\frac{\partial {C_{0}}^{k}}{\partial z}=0\to {C_{1}}^{k}={C_{-1}}^{k} \end{array}$$

(A8) $$\begin{array}{lll} z & = & L:\frac{\partial {C_{n+1}}^{k}}{\partial z}=0\to {C_{n+2}}^{k}={C_{n}}^{k} \end{array}$$

The initial condition for inspiration is defined as *C*(*z*,0) = *C*_j_^0^ = 0, while for expiration, the last axial concentration profile of the inhalation period is considered as initial condition. Here, *n* is the node before the last node and the node at *z* = 0 represents the mouth. The matrix Equation (A1) can then be solved numerically considering the defined boundary and initial conditions.

## Notations

### Nomenclature

**Table:**

*A*_c,aw_ (*z*)	Cross-sectional area of airway compartment at location *z* (cm^2^)
*A*_c,1_	Cross-sectional area of the airways at generation 17 (cm^2^)
*A*_c,A_	Total cross-sectional area of alveolar compartment (cm^2^)
*C*_CO_	Concentration of gaseous CO in the respiratory tract (ppb)
*C*_ACO_	Alveolar CO concentration (ppb)
*C*_tiss_	Airway tissue CO concentration (ppb)
*C*_amb_	Ambient air CO concentration (ppb)
*C*_CO,ET_	End-tidal CO concentration (ppb)
*D'*_awCO_	Diffusing capacity of CO in the airway per unit axial distance (pl.s^–1^.ppb^–1^.cm^–1^)
*D*_awCO_	Total diffusing capacity of CO in the airway (pl.s^–1^.ppb^–1^)
*D'*_ACO_	Diffusing capacity of CO in the alveoli per unit axial distance (pl.s^–1^.ppb^–1^.cm^–1^)
*D*_ACO_	Total diffusing capacity of CO in the alveolar region (pl.s^–1^.ppb^–1^)
*D*_CO,air_	Molecular diffusivity of CO in air (cm^2^.s^–1^)
*J'*_awCO_	Maximum volumetric flux of CO from the airways per unit axial distance (pl.s^–1^.cm^–1^)
*J*_awCO_	Total maximum volumetric flux of CO from the airways (pl/s)
*J'*_ACO_	Maximum volumetric flux of CO from the alveoli per unit axial distance (pl.s^–1^.cm^–1^)
*J*_ACO_	Total maximum volumetric flux of CO from the alveoli (pl/s)
*L*	Total length of respiratory tract in trumpet model (27.20 cm)
*N*_alv_ (*z*)	Number of alveoli per unit axial distance
*N*_t_	Total number of alveoli
*N*_max_	Maximum number of alveoli at any axial position
$\overset{\cdot }{V}$_I_	Volumetric flow rate of air during inhalation (IFR; ml/s)
$\overset{\cdot }{V}$_E_	Volumetric flow rate of air during exhalation (EFR; ml/s)
*V*	Average of inhaled/exhaled volume (ml)
*z*	Axial position in the lung (cm)

### Unit conversion

1 mmHg = 10^9^/760 ppb

1 mmHg = 10^2^/760 %

1 ppb = 760.10^–9^ mmHg

1 ppm = 1,000 ppb

1 atm = 760 mmHg = 760 Torr
